# Lack of Casein Kinase 1 Delta Promotes Genomic Instability - The Accumulation of DNA Damage and Down-Regulation of Checkpoint Kinase 1

**DOI:** 10.1371/journal.pone.0170903

**Published:** 2017-01-26

**Authors:** Yoshimi Endo Greer, Bo Gao, Yingzi Yang, Andre Nussenzweig, Jeffrey S. Rubin

**Affiliations:** 1 Laboratory of Cellular and Molecular Biology, National Cancer Institute, Bethesda, Maryland, United States of America; 2 Women’s Malignancies Branch, National Cancer Institute, Bethesda, Maryland, United States of America; 3 Genetic Disease Research Branch, National Human Genome Research Institute, Bethesda, Maryland, United States of America; 4 Laboratory of Genome Integrity, National Cancer Institute, Bethesda, Maryland, United States of America; German Cancer Research Center, GERMANY

## Abstract

Casein kinase 1 delta (CK1δ) is a conserved serine/threonine protein kinase that regulates diverse cellular processes. Mice lacking CK1δ have a perinatal lethal phenotype and typically weigh 30% less than their wild type littermates. However, the causes of death and small size are unknown. We observed cells with abnormally large nuclei in tissue from *Csnk1d* null embryos, and multiple centrosomes in mouse embryo fibroblasts (MEFs) deficient in CK1δ (MEF^*Csnk1d null*^). Results from γ-H2AX staining and the comet assay demonstrated significant DNA damage in MEF^*Csnk1d null*^ cells. These cells often contain micronuclei, an indicator of genomic instability. Similarly, abrogation of CK1δ expression in control MEFs stimulated micronuclei formation after doxorubicin treatment, suggesting that CK1δ loss increases vulnerability to genotoxic stress. Cellular levels of total and activated checkpoint kinase 1 (Chk1), which functions in the DNA damage response and mitotic checkpoints, and its downstream effector, Cdc2/CDK1 kinase, were often decreased in MEF^*Csnk1d null*^ cells as well as in control MEFs transfected with CK1δ siRNA. Hydroxyurea-induced Chk1 activation, as measured by Ser345 phosphorylation, and nuclear localization also were impaired in MEF cells following siRNA knockdown of CK1δ. Similar results were observed in the MCF7 human breast cancer cell line. The decreases in phosphorylated Chk1 were rescued by concomitant expression of siRNA-resistant CK1δ. Experiments with cycloheximide demonstrated that the stability of Chk1 protein was diminished in cells subjected to CK1δ knockdown. Together, these findings suggest that CK1δ contributes to the efficient repair of DNA damage and the proper functioning of mitotic checkpoints by maintaining appropriate levels of Chk1.

## Introduction

Casein kinase 1 delta (CK1δ) is an evolutionarily conserved serine/threonine kinase that participates in diverse cellular processes, including vesicle trafficking, chromosome segregation, circadian rhythm, Wnt signaling, neurite outgrowth and ciliogenesis [1–6]. Several studies have demonstrated an important role for CK1δ in DNA repair and cell cycle regulation [7]. Hrr25, the CK1δ ortholog in budding yeast, was first identified when one of its mutants was associated with a deficiency in DNA repair [8]. Subsequent work indicated that Hrr25 contributes to the transcriptional response to DNA damage [9], and others reported that mutation of Hrr25 resulted in a mitotic checkpoint defect [10]. CK1 homologs are also required for a mitotic checkpoint in the fission yeast *S*. *pombe* [11].

In mammalian cells, CK1δ localizes to microtubules, the Golgi apparatus, cytoplasmic vesicles and the centrosome as well as the mitotic spindle apparatus [5, 6, 12, 13], consistent with a potential involvement in mitotic checkpoint regulation. CK1δ has been implicated in the control of p53 stability, providing a mechanism for disruption of the mitotic checkpoint when CK1δ activity is abrogated. CK1δ phosphorylates p53 at Thr18 [14, 15], thereby inhibiting its association with Mdm2, the E3 ubiquitin ligase that mediates turnover of p53[16]. CK1δ also directly phosphorylates Mdm2, which facilitates its degradation via SCF^beta-TRCP^ ubiquitin-ligase, consequently promoting the stability and function of p53[17, 18]. Thus, the deficiency in DNA repair that has been correlated with a mitotic checkpoint defect in yeast has a counterpart in mammalian cells, and has been attributed to dysregulation of p53 turnover.

Checkpoint kinase 1 (Chk1), a serine/threonine-specific protein kinase, is another primary mediator of the DNA damage response (DDR) and mitotic checkpoints [19–21]. Initially Chk1 was recognized as a regulator of the G2/M checkpoint, and was subsequently shown to have additional roles in replication fork stability, replication origin firing and homologous recombination, all of which are critical processes for DNA repair [22, 23]. Genotoxic stresses such as DNA replication stalling by hydroxyurea (HU), ionizing radiation and ultraviolet light result in formation of single strand DNA breaks that attract ATR (Ataxia telangiectasia and Rad3-related protein) [24]. ATR, in turn, activates Chk1 by phosphorylation at Ser317 and Ser345 [25]. Activation of ATM (Ataxia telangiectasia mutated) by double strand DNA breaks can also lead to Chk1 activation [25]. Activated Chk1 phosphorylates the Wee1 kinase and the Cdc25 phosphorylase, activating the former while inhibiting the latter, both of which control the phosphorylation of Cdc2/cyclin-dependent kinase CDK1. The net effect is phosphorylation of Cdc2/CDK1 at Tyr15, which inhibits its activity, and thereby enables G2 or S phase arrest [26]. Phosphorylation at Ser345 is critical for a conformational change in Chk1 that is required for its kinase activity [27]. In addition, phosphorylation at Ser345 promotes its nuclear retention [28], where Chk1 plays a direct role in the DDR. Inhibition of Chk1 enables CDK1/2 to abrogate cell cycle arrest before the cells complete repair, driving the damaged S phase cells into G2 and G2 cells through an aberrant mitosis (e.g., mitotic catastrophe) [29]. Because such events often lead to cell death, Chk1 inhibitors have been tested in clinical trials to assess their ability to potentiate the killing effects of established antimetabolite agents in cancer therapy [30, 31].

A recent study showed that Chk1 phosphorylates rat and human CK1δ at the C-terminus, and that site-specific phosphorylation of CK1δ by Chk1 modulates the activity of CK1δ *in vitro* [32], indicating a functional regulation of CK1δ by Chk1. Here, we report that lack of CK1δ leads to gross nuclear defects and accumulation of DNA damage in mammalian cells, and these defects are associated with a reduction in activated Chk1 and increased turnover of Chk1. Our findings indicate a previously unrecognized regulation of Chk1 by CK1δ that has a significant effect on the response to DNA damage and the presumed functioning of mitotic checkpoints.

## Materials and Methods

### Chemicals

Doxorubicin (cat#D1515), hydroxyurea (cat#H8627), DAPI ([4’, 6-diamidino-2-phenylindole]dihydrochloride, cat# D9542), cycloheximide (cat#C-7698) were purchased from Sigma-Aldrich (St. Louis, MO, USA). PF670462 (cat#3316) and LH846 (cat#4896) were purchased from TOCRIS (Bristol, BS11 0QL, United Kingdom). Propidium iodide was from Life Technologies (cat#P3566).

### Tissue isolation and genotyping

This study was carried out in accordance with the recommendations in the *Guide for the Care and Use of Laboratory Animals of the National Institutes of Health*. Our protocol was approved by the NCI-Bethesda Animal Care and Use Committee (Protocol Number: LCMB-029). Mice were obtained from The Jackson Laboratory (Bar Harbor, ME). Pregnant female mice were euthanized via inhalation of carbon dioxide. Isolation of retinal tissue from mouse embryos for immunostaining was performed as previously reported [6]. Isolation of mouse embryonic fibroblast (MEF) cells from embryos carrying two copies of a *Csnk1d* floxed allele (B6.129S4-*Csnk1dtm1Drw*/J, stock number: 010487) [33] and generation of *Csnk1d* null (MEF^*Csnk1d null*^) or control MEF (MEF^*Ctl*.^) cells were also performed as previously described [6]. For genotyping of neonatal mice, tail tissues from pups were cut out and incubated with 500 μl of tail lysis buffer (50mM Tris pH 8.0, 200mM NaCl, 5mM EDTA pH 8.0, 0.2% SDS) supplemented with protein K (10mg/ml) at 50°C overnight. Next day, 500 μl of Phenol: Chloroform: IsoamylAlcohol (24:24:1, Invitrogen Cat#15593–031) were added and mixed well, then centrifuged at 20,817 × *g* for 5 min at room temperature (RT). Upper layer was transferred to new tube, then mixed with 1 ml of 100% ethanol, followed by centrifugation at 20,817 × *g* for 5 min at RT. Supernatant was discarded and genomic DNA pellet was washed with 70% ethanol by 1 min centrifugation. Genomic DNA was dissolved with 100–200 μl ultrapure water and used for polymerase chain reaction (PCR). PCR protocol indicated at The Jackson Laboratory website was used for genotyping. Briefly, PCR protocol was 95°C for 5 min, 95°C/60°C /72°C (45 sec each) for 35 cycles, followed by 72°C for 10 min and 15°C for infinite time. PCR primer sequences were 9903: GCT GAG GCC AAT AAA GAG TCT GTC ACA TG (common), 9905: GTA GTT TGC CTG ATG AAA CAG GAG C (WT forward), 9906: CAG CCC TAG TTA TCG TAA CAT CG (mutant forward). PCR product size of mutant was 612 bp, heterozygous was 612 bp and 235 bp, wild type was 235 bp.

### Cell Culture

MEF cells and HEK293 cells were maintained in Dulbecco’s modified Eagle's medium (DMEM) supplemented with 10% fetal bovine serum (FBS), 100 U/ml penicillin and 100 μg/ml streptomycin (pen/strep) in a 5% CO_2_ humidified 37°C cell culture incubator. MCF7 cells were maintained in RPMI 1640 supplemented with 10% FBS and penicillin/streptomycin.

### Flow Cytometry

Cells were grown in culture dishes, collected and resuspended in phosphate-buffered saline (PBS) at a concentration of 1–2 x 10^7^ cells/ml. One ml of cell suspension was pipetted into a Falcon 12x75 mm polystyrene tube with snap cap and placed in an ice bath for 15 min. While vortexing the tube on medium speed, 1 ml ice-cold 70% ethanol was added gradually, and samples were stored at 4°C overnight for fixation. Cells fixed in ethanol were spun down by centrifugation (300 g, 5 min), and the ethanol was removed. RNAse (cat#R-5503, Sigma-Aldrich, 100 units in 100 μl) was added to each tube and incubated at 37°C for 20 min, followed by the addition of 500 μl propidium iodide (50 μg/ml in PBS). Cells were incubated at 4°C in the dark for at least 30 min before flow cytometry. Cell cycle analysis was performed with BD Calibur at NCI FACS core facility (https://ostr.cancer.gov/resources/ncr/Flow%20Cytometry) using BD CellQuest software (BD Biosciences) and ModFit software (Verity Software House, http://www.vsh.com).

### Comet assay

The comet assay was performed with CometAssay^®^ Kit (cat# 4250-050-K, Trevigen, Gaithersburg, MD, USA) according to the manufacturer’s protocol. Briefly, Comet LM Agarose (molten at 37°C, 500 μl) and cells (suspended in PBS [Ca^2+^ and Mg^2+^ free] at 1 x 10^5^/ml, 50 μl) were combined, and 50 μl of the mixture was immediately placed onto a comet slide. After 30 min incubation at 4°C in the dark, slides were immersed in 4°C lysis solution for 2 h at RT. Subsequently, slides were immersed in freshly prepared alkaline unwinding solution (pH > 13) for 1 h at RT in the dark. Using a CometAssay^™^ ES unit, electrophoresis was performed with Alkaline Electrophoresis Solution at 21 volts for 1 h. After draining the excess electrophoresis solution, slides were immersed twice in dH_2_O, once in 70% ethanol for 3 min, and then dried at 37°C for 15 min. SYBR Green solution (cat#50512, Lonza, Basel, Switzerland) was added to the dried agarose samples, which were stained for 30 min at RT in the dark. The comet image was captured with epifluorescent microscopy and tail moment was analyzed with OpenComet software (http://www.cometbio.org).

### Cell viability assay

MEF^*Ctl*.^ cells were seeded at 5,000 cells/well in 96-well plate on the day before treatment. PF670462-mediated cytotoxcity was assessed by the MTS assay using Cell Titer 96^®^ AQueous One Solution Cell Proliferation Assay (cat#G3581, Promega, Madison, WI, USA) according to the manufacturer’s protocol ([34]). All MTS measurements were performed in triplicates and each experiment was carried out four times.

### siRNA reagents and transfection

siRNA transfection experiments were performed with Lipofectamine RNAi Max (cat#13778–075, ThermoFisher Scientific, Grand Island, NY) according to the manufacturer's protocol. For CK1δ knockdown in MEF cells, Mm_Csnk1d_1 FlexiTube siRNA (siCK1δ _Q1, Qiagen) and Mm_Csnk1d_2 FlexiTube siRNA (siCK1δ _Q2, Qiagen) were used. For CK1δ knockdown in human cells, Hs_CSNK1D_6 FlexiTube siRNA (siCK1δ _Q6, Qiagen,) and CSNK1D siRNA from Ambion (siCK1δ_Am) were used (for sequence information, see S1 Table). All Stars Negative Control siRNA (siNeg, Qiagen) served as the negative control. The effects of siRNA treatment were analyzed 48–72 h after transfection.

### Recombinant DNA and transfection

pcDNA3.3 6xMyc-mouse CK1δ wild type (WT) and a kinase-dead (K38A) derivative were previously described [5, 6]. For DNA transfection in MEF cells, Lipofectamine LTX with plus reagent (cat#15338–030, ThermoFisher Scientific) was used according to the manufacturer’s protocol.

### Antibodies used for immunoblotting and immunostaining

See S1 Table.

### Immunoblotting

Eighty to 90% confluent monolayers that had been seeded in 6- or 12-well cell culture plates were rinsed twice with PBS, lysed and processed for SDS-PAGE and western blot analysis as previously described [5]. For immunoblot analysis to verify siRNA knockdown of endogenous proteins, cells that had been transfected with siRNA reagents were harvested 48–72 h after transfection. To detect γ-H2AX, cells were lysed with cell lysis buffer and spun down at 20,817 × *g* for 15 min at 4°C. Supernatant and pellets were separated, and supernatant was used for detection of other proteins (HSC70, CK1δ), while pellets were resuspended with 15 μl of 0.2N HCl and incubated on ice for 15 min. Pellet samples were neutralized with 2 μl of 2M NaOH, then resuspended with lysis buffer and protein concentration was determined (protein assay dye reagent, Bio-Rad, cat# 5000006) prior to SDS-PAGE and immunoblotting for γ-H2AX.

### Immunoprecipitation

HEK293 cells were harvested with IP buffer (20 mM Tris-HCl, pH 8.0, 10 mM EDTA, 1 mM EGTA, 150 mM NaCl, 0.2% Triton-X, 0.2% Nonidet P-40), supplemented with protease inhibitors (complete Mini EDTA-free, cat#11836170001, Sigma-Aldrich). Cell lysates were homogenized by passing through 23-gauge syringe needles, and centrifuged at 20,817 × g for 15 min at 4°C. Supernatant was transferred to another tube, and protein concentration was determined by Bio-Rad protein assay dye reagent. Cell lysates (1 mg) was incubated with 1 μg of mouse IgG and 20 μl of protein A/G Plus-agarose for 2 h at 4°C, followed by centrifugation at 20,817 × g for 10 min. In parallel, 1 μg of FLAG antibody and 20 μl of protein A/G-agarose were combined in 1 ml IP buffer and incubated for 2 h at 4°C with rotation. After 2 h, centrifugation at 956 × g for 5 min. Pre-cleared lysate and target antibody/protein A/G-agarose mixture were combined and incubated overnight at 4°C with rotation. After 18 h, protein A/G beads were washed with IP buffer three times, and 30 μl of 2x Laemmli buffer (supplemented with 5% beta-mercaptoethanol) was added. The samples were heated at 95°C for 10 min, and subjected to SDS-PAGE followed by western blotting.

### Immunofluorescent analysis

MEF cells cultured on 12 mm diameter glass coverslips (Fisher, Pittsburgh, PA, USA) were first washed once with PBS, then once with PHEM (60 mM Na-Pipes, 25 mM Na-HEPES, 10 mM Na-EGTA, and 2 mM MgCl_2_, pH 6.9). Cells were fixed with freshly prepared 3.7% formaldehyde and 1% Triton X-100 in PHEM supplemented with Taxol (10 mM) for 10 min at RT. Cells were then washed with PBS, and blocked with 6% BSA in PBS for 30 min at RT. Then primary antibody/antibodies was/were added with blocking solution, and samples were incubated for 1 h at 37°C or overnight at 4°C. After washing three times with PBS, samples were incubated with secondary antibody reagent(s) and DAPI in blocking solution for 45 min at RT. After washing three times with PBS, coverslips were mounted on glass slides (VWR Scientific) using ProLong Gold Antifade reagent (cat#P36930, Invitrogen). To visualize gamma-tubulin, cells were first washed once with PBS, then with PHEM, followed by treatment with PHEM containing 0.19 M NaCl, 1% Saponin, 10 μM Taxol, and 0.1% DMSO for 5 min at RT to permeabilize cells and stabilize tubulin. Cells were then immersed in cold 100% MeOH at −30°C for 10 min, rehydrated by rinsing three times with PBS, and treated with blocking solution for 30 min at 37°C. Primary and secondary antibodies were added as described above.

### Cell imaging

Fluorescent images were collected with a laser-scanning confocal microscope (510 LSCM), and a 63x objective (Carl Zeiss Inc.). Zeiss LSM image browser Version 4.0.0.157 was used for image processing, and composite figures were prepared with Adobe PhotoShop CS3 Ver 10.0.1 (Adobe Systems Inc.). Fluorescence intensity was measured by ImageJ software (https://www.unige.ch/medecine/bioimaging/files/1914/1208/6000/Quantification.pdf).

### Protein stability

MEF^*Ctl*.^ cells were transfected with siNeg or siCK1δ, and 40, 42, 46 and 47 h later, cycloheximide (CHX) was added at a final concentration of 10 μg/ml to the cells for 8, 6, 2 and 1 h treatment, respectively. Forty-eight h after siRNA transfection, cells (including ones that did not receive CHX) were harvested and western blot analysis was performed.

### Statistical analysis

The significance of differences in data was determined with Student’s *t*-test unless otherwise noted. The differences were considered to be significant when P value was less than 0.05. For B in S4 Fig, Fisher’s exact test was used.

## Results

### Absence of CK1δ from mouse cells results in abnormal nuclear phenotypes indicative of genomic instability

**Fig 1:**
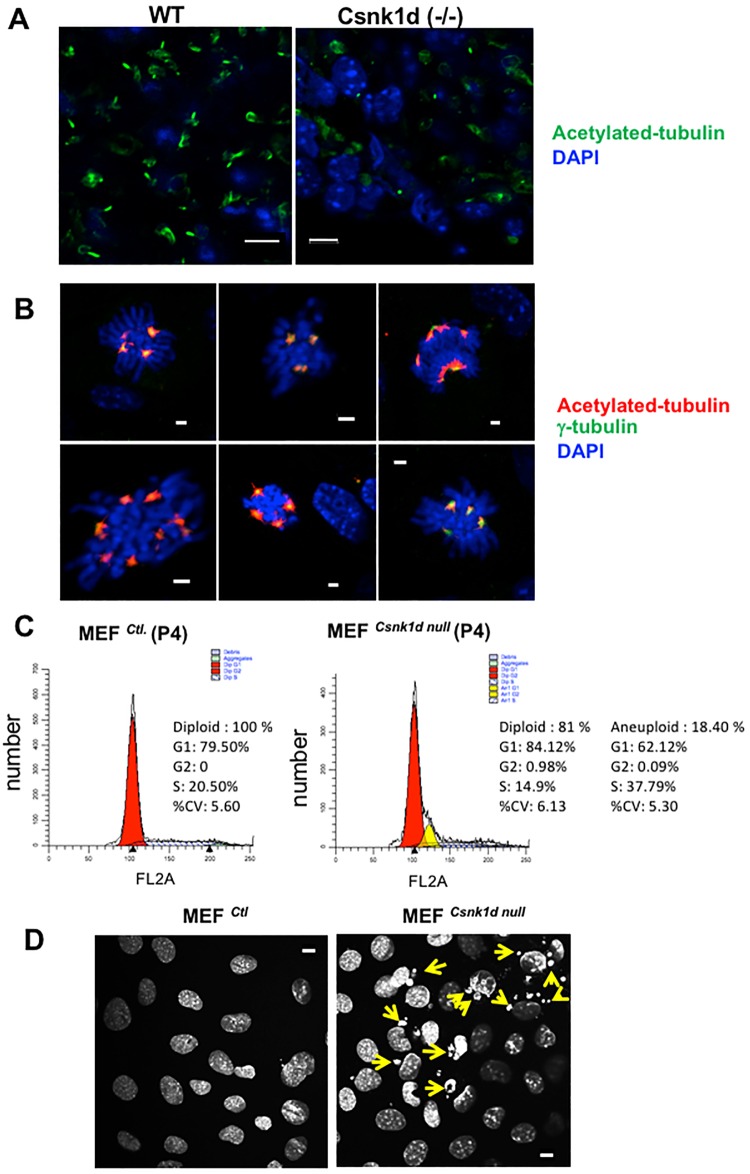
MEF^*Csnk1d null*^ cells exhibit abnormal nuclear phenotypes indicative of genomic instability. (A) Retinal tissue from wild type and *Csnk1d* null E18.5 mouse embryos stained for DNA and acetylated tubulin. Representative micrographs, stained with DAPI (blue) to visualize nuclei and antibody to acetylated tubulin (green) to detect cilia. Bars, 5 μm. (B) MEF^*Csnk1d null*^ cells (passage 4) with multiple centrosomes. Cells were stained with DAPI (blue), and antibodies to acetylated tubulin (red) and γ-tubulin (green), the latter was a centrosomal marker. Yellow indicates overlap of red and green stains. Bars, 2 μm. (C) Flow cytometric analysis of MEF^Ctl.^ and MEF^*Csnk1d null*^ cells demonstrated aneuploidy in the latter. Percentages of diploid and aneuploid cells in the different phases of the cell cycle are indicated. Graphs show analysis of live cells; see S2 Fig for inclusion of sub G0/G1 population. (D) MEF^*Csnk1d null*^ cells contain micronuclei. MEF^*Ctl*.^ and MEF^*Csnk1d null*^ cells (both P4) were stained with DAPI. Arrows indicate micronuclei. Bars, 10 μm.

Mice that were homozygous for a *Csnk1d* allele previously shown to disrupt expression of CK1δ protein have a perinatal lethal phenotype, and weigh 30–50% less than heterozygous littermates [33] (S1 Fig). Cellular or molecular defects accounting for these traits have yet to be described. During our earlier work with *Csnk1d* null mice [6], we noticed that retinal cells from 18.5 d *Csnk1d* null embryos often had grossly enlarged nuclei (Fig 1A). Analysis of MEF^*Csnk1d null*^ cells also revealed a small fraction (5–10%) of cells with large, DAPI-stained nuclei that contained three or more centrosomes (Fig 1B), features not seen in MEF^*Ctl*.^ cells. Flow cytometric analysis indicated that 18.4% of MEF^*Csnk1d null*^ cells exhibited aneuploidy, which was absent from MEF^*Ctl*.^ cells (Fig 1C). Micronuclei also were detected in many MEF^*Csnk1dnull*^ cells, particularly in the early passages (P2-P5) (Fig 1D, P4 cells), when there also was evidence of increased cell death in MEF^*Csnk1d null*^ cells (S2 Fig). Micronuclei are primarily comprised of chromosomes or chromosomal fragments enclosed within a nuclear membrane [35], and are indicative of defective mitosis (reviewed in [36]). The presence of grossly enlarged nuclei, multiple centrosomes, flow cytometric evidence of aneuploidy and micronuclei in many cells lacking CK1δ strongly suggest that deficiency in the expression of this protein contributes to genomic instability.

### Loss of CK1δ leads to accumulation of DNA damage

**Fig 2:**
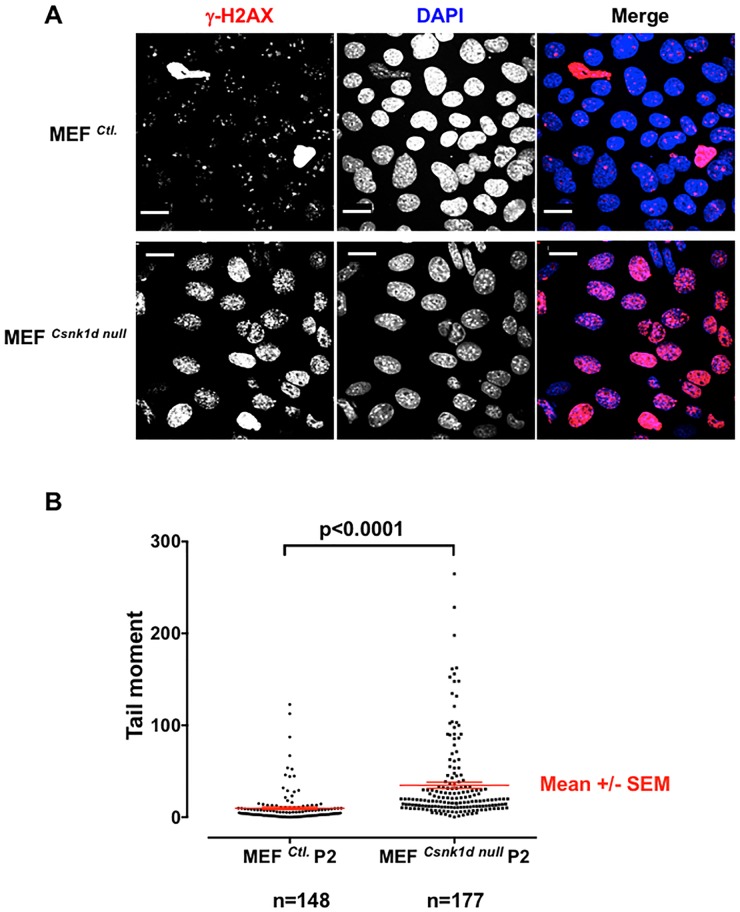
MEF^*Csnk1d null*^ cells have more DNA damage than MEF^Ctl.^ cells. (A) DNA damage response in MEF^*Ctl*.^ and MEF^*Csnk1d null*^ cells. MEF^*Ctl*.^ and MEF^*Csnk1d null*^ cells (P2) stained with antibody to γ-H2AX (red), a marker for double strand breaks, and DAPI (blue). Bars, 20 μm. (B) Comet assay demonstrated greater amount of DNA damage in MEF^*Csnk1d null*^ cells. Size of tail moment corresponds to extend of DNA damage in the cell. “n” indicates the number of cells analyzed. Data are from one representative experiment among three independent experiments.

**Fig 3:**
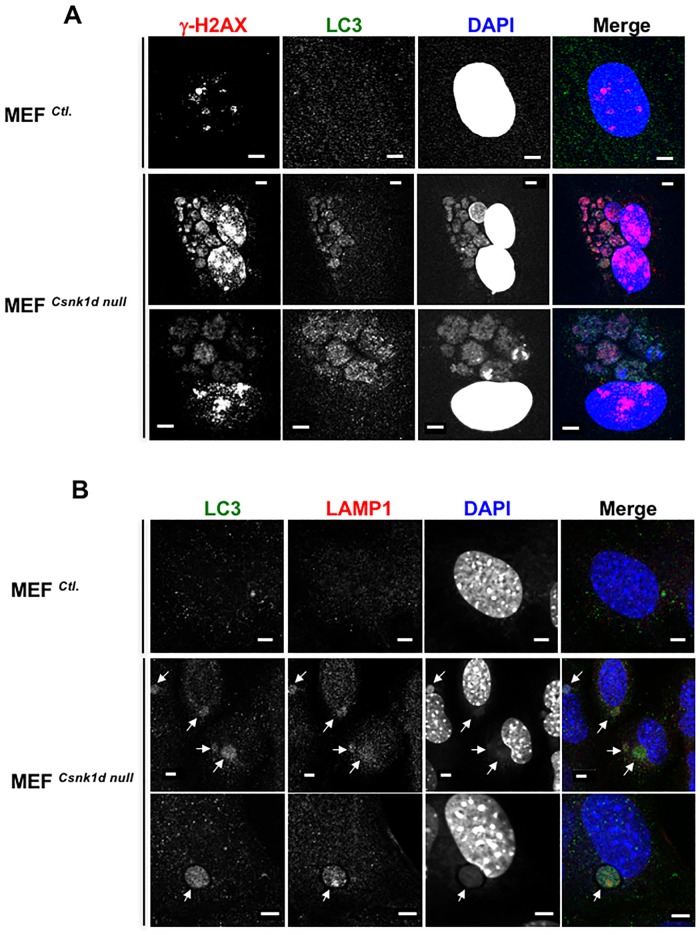
Micronuclei in MEF^*Csnk1d null*^ cells are characterized by DDR and autophagosomal markers. (A) MEF^*Ctl*.^ and MEF^*Csnk1d null*^ cells (both, P5) were stained with DAPI (blue), and antibodies to γ-H2AX (red) and the autophagosomal marker, LC3 (green). Bars, 5 μm. (B) MEF^*Ctl*.^ and MEF^*Csnk1d null*^ cells (both, P7) were stained with DAPI (blue), and antibodies to the autophagosomal markers, LC3 (green) and LAMP1 (red). Bars, 5 μm.

Because genomic instability often is associated with DNA damage, we sought evidence of an increased DNA damage response (DDR) in cells deficient in CK1δ by immunostaining with γ-H2AX antibody [37]. Only a few MEF^*Ctl*.^ cells in monolayer culture showed signs of a DDR in the absence of exposure to a genotoxic agent. In marked contrast, MEF^*Csnk1d null*^ cells exhibited massive γ-H2AX staining, indicating that a lack of CK1δ induced a strong DDR (Fig 2A). A similar result was observed following siRNA-mediated knockdown of CK1δ in MCF7 cells (S3 Fig), a human breast cancer cell line homozygous for wild type p53 [38]. Quantitative analysis of DNA damage with the single cell alkaline comet assay confirmed that there was a highly significant accumulation of DNA damage in MEF^*Csnk1d null*^ cells compared to MEF^*Ctl*.^ cells (Fig 2B). Substantial γ-H2AX staining also was detected in the DAPI-positive micronuclei commonly seen in MEF^*Csnk1d null*^, but not MEF^*Ctl*.^ cells (Fig 3A). Treatment of MEF^*Ctl*.^ cells with PF670462, a CK1δ /ε inhibitor, also increased γ-H2AX staining and micronuclei formation, while decreasing cell viability (S4 Fig). This implied that CK1δ kinase activity is involved in the DNA damage response and genomic stability.

Further analysis revealed that the micronuclei in MEF^*Csnk1d null*^ cells stained positively for LC3 (microtubule-associated protein 1A/1B-light chain 3), which is recruited to autophagosomal membranes during the process of autophagy (Fig 3A). The lysosomal marker LAMP1 also was detected in the micronuclei (Fig 3B), reinforcing the idea that they were destined to undergo nucleophagy, an autophagic mechanism to eliminate aberrant nuclear material [39].

**Fig 4:**
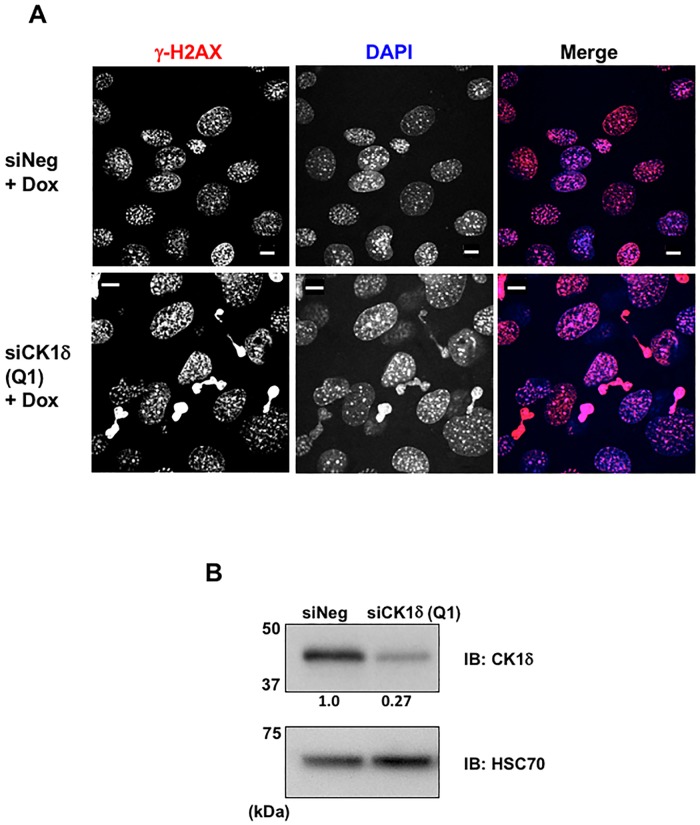
Doxorubicin stimulated the formation of γ-H2AX laden micronuclei in cells treated with CK1δ siRNA. (A) MEF^*Ctl*.^ cells (P5) were transfected with negative control siRNA or CK1δ siRNA (Q1), and 67 h later treated with doxorubicin (5 μM) for 5 h. Cells were subsequently fixed and stained with γ-H2AX antibody (red) and DAPI (blue). Bars, 10 μm. (B) Western blot analysis of MEF^*Ctl*.^ cells treated with siRNA reagents as described in (A) Relative band intensity of CK1δ immunoblot (normalized with HSC70 loading control) is shown below the panel.

To further investigate the impact of reduced CK1δ expression on the accumulation of DNA damage, we examined γ-H2AX staining in MEF^*Ctl*.^ cells exposed to a DNA damaging agent. MEF^*Ctl*.^ cells were transfected with either control siRNA or CK1δ siRNA, and doxorubicin was used to trigger DNA damage. MEF^*Ctl*.^ cells that had been transfected with either CK1δ or negative control siRNA exhibited substantial γ-H2AX nuclear staining after exposure to doxorubicin. However, only the cells that were treated with CK1δ siRNA contained micronuclei, and these structures were intensely stained with γ-H2AX antibody (Fig 4A). The efficacy of CK1δ siRNA knockdown was confirmed in Fig 4B. These results demonstrated that CK1δ loss undermined the ability of the cells to respond to genotoxic stress.

### Chk1 protein level and activation are reduced in the absence of CK1δ

**Fig 5:**
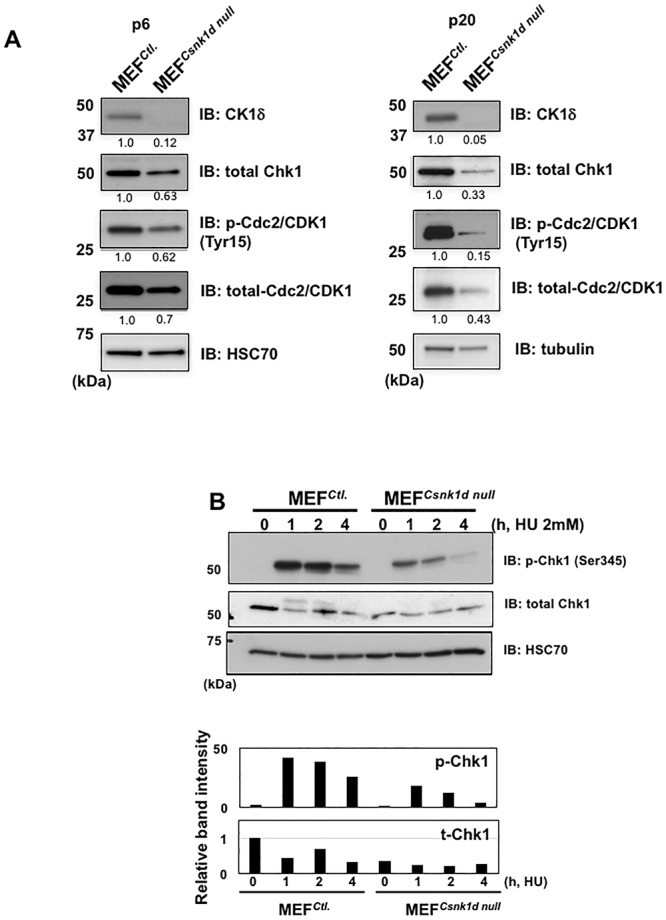
MEF^*Csnk1d null*^ cells have lower levels of total and phosphorylated Chk1 and Cdc2/CDK1 than MEF^Ctl.^ cells. (A) Western blot analysis of total Chk1, total and phosphorylated (Tyr15) Cdc2/CDK1, and CK1δ in MEF^*Ctl*.^ and MEF^*Csnk1d null*^ cells from two sets of cells with different passage number (left, P6; right, P20). Band intensity relative to the HSC70 or tubulin loading control is indicated directly below each band. (B) Time course of Chk1 activation, as indicated by Ser345 phosphorylation, in the same passage of MEF^*Ctl*.^ and MEF^*Csnk1d null*^ cells treated with hydroxyurea (HU). Histogram shows the band intensity of p-Chk1and total Chk1 relative to the HSC70 loading control. Band intensity of MEF^*Ctl*.^ cells not exposed to HU was defined as 1.0. (C) MEF^*Ctl*.^ (P9) cells were transfected with siRNA, and 46 h later cells were treated with HU for 1.5 h, followed by western blotting. Data in left panel are from one representative experiment. Histograms to the right show relative band intensities of p-Chk1, total Chk1 and CK1δ from two independent experiments. Band intensity of MEF^*Ctl*.^ cells transfected with siNeg and not exposed to HU was defined as 1.0.

The accumulation of DNA damage in cells lacking CK1δ suggested that they had a defect in the DDR mechanism. Because Chk1 is an important mediator of the DDR that binds CK1δ and modulates its activity [32], we tested the hypothesis that Chk1 is regulated by CK1δ. Our analysis revealed a variable, though often sharp decline in the amount of Chk1 in MEF^*Csnk1d null*^ cells compared with MEF^*Ctl*.^ cells (Fig 5A). Moreover, we observed in MEF^*Csnk1d null*^ cells a decrease in phosphorylated and total Cdc2/CDK1 (Fig 5A), an important downstream effector of Chk1 that mediates the G2/M mitotic checkpoint when it is phosphorylated at Tyr15 (reviewed in [40]). The decrease in Chk1, phosphorylated and total Cdc2/CDK1 was evident both at lower (P6) and higher (P20) cell passages. These findings suggested that the absence of CK1δ disrupts the Chk1-dependent G2/M checkpoint.

**Fig 6:**
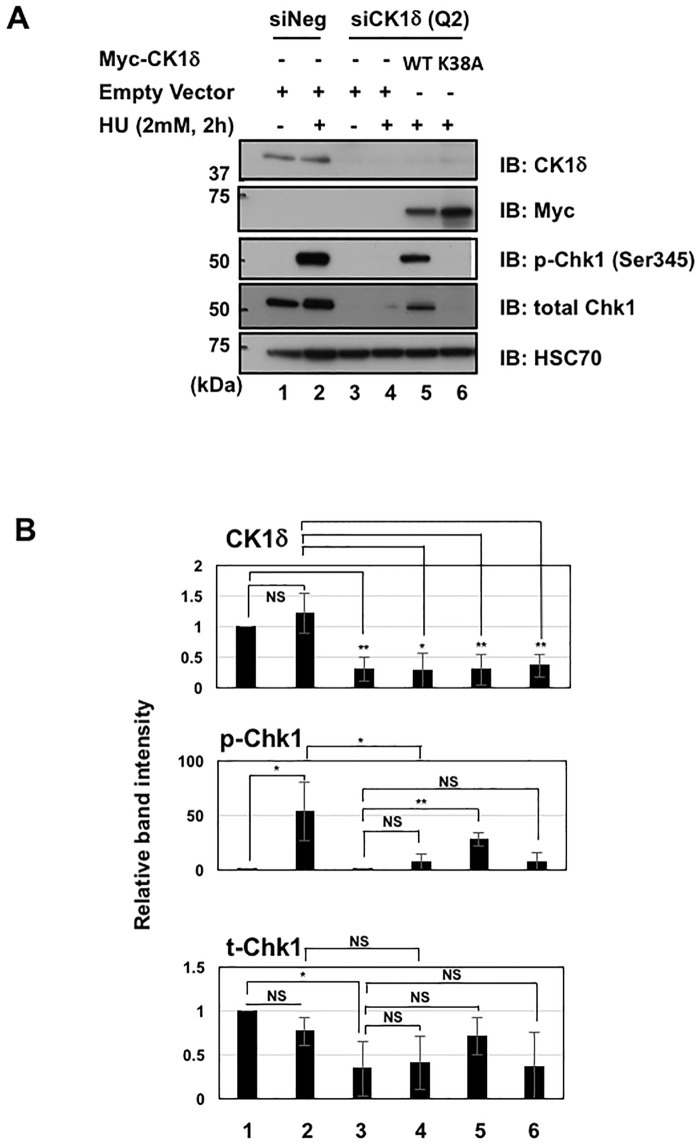
Analysis of total and phosphorylated Chk1 in MEF^Ctl.^ cells after HU treatment, following CK1δ knockdown with or without subsequent expression of siRNA-resistant CK1δ constructs. (A) MEF^*Ctl*.^ cells were transfected with control siRNA (siNeg) or siRNA targeting CK1δ (siCK1δ Q2). Twenty-four hours later, cells were transfected with the indicated DNA constructs (pcDNA 3.3 was the empty vector control). Seventy hours later, cells were treated with HU for 2 h and subjected to western blotting. Data are from one representative experiment among 3 independent experiments. (B) Statistical analysis of band intensity shown in (A). Relative band intensity of CK1δ, p-Chk1 and total Chk1 was analyzed in 3 independent experiments and shown as mean +/- SD. *p<0.05, **p<0.01, NS = not significant.

Next, we investigated the possibility that Chk1 activation induced by DNA replicative stress is perturbed in the absence of CK1δ. Treatment of cells with hydroxyurea (HU) normally stimulates replicative stress and activates Chk1, via phosphorylation at Ser345 [41]. The time-dependent, HU-mediated induction of Chk1 phosphorylation at Ser345 appeared to be diminished in MEF^*Csnk1d null*^ cells compared with MEF^*Ctl*.^ cells (Fig 5B). Furthermore, knockdown of CK1δ in MEF^*Ctl*.^ cells also blocked the induction of Chk1 phosphorylation by HU (Fig 6). A correlation of CK1δ siRNA knockdown with decreased basal levels of Chk1 and HU-induced levels of phosphorylated Chk1 also was seen in MCF7 cells (S5 Fig). Importantly, concomitant transfection of MEF^*Ctl*.^ cells with an siRNA-resistant, wild type (WT) CK1δ expression vector partially restored Chk1 activation in response to HU (Fig 6). Transfection with an siRNA-resistant vector encoding a kinase-dead (K38A) CK1δ derivative did not rescue Chk1 activation, implying that CK1δ kinase activity contributed to the regulation of Chk1 (Fig 6). To further examine the role of CK1δ kinase activity in Chk1 phosphorylation, MEF^*Ctl*.^ cells were pre-incubated for 1 h with a CK1δ/ε inhibitor, either PF670462 or LH846, and subsequently treated with HU. However, neither of these compounds inhibited HU-induced phosphorylation of Chk1 (S6 Fig). This result raises questions about the function of CK1δ kinase activity in the regulation of Chk1 phosphorylation that will be addressed in the Discussion.

### Nuclear distribution of Chk1 is inhibited when CK1δ expression is suppressed

**Fig 7:**
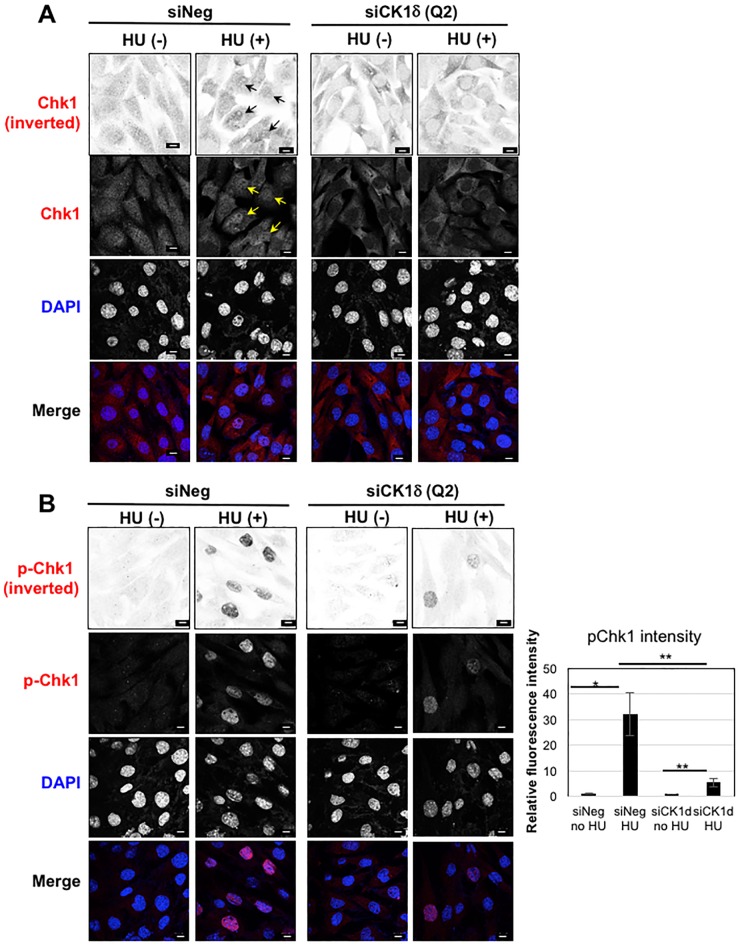
Nuclear distribution of Chk1 and p-Chk1 in MEF^Ctl.^ cells is inhibited by siRNA knockdown of CK1δ. Cellular distribution of (A) Chk1 and (B) phosphorylated Chk1. MEF^*Ctl*.^ cells were transfected with negative control (siNeg) or CK1δ siRNA (siCK1δ), and 70 h later culture fluid was removed and fresh medium added with or without hydroxyurea (HU, 2mM final concentration) for 2 h incubation. Arrows indicate accumulation of Chk1 in the nucleus. Bars, 10 μm. Histogram shows statistical analysis of p-Chk1 intensity in the nucleus. Data is shown as mean +/- SD. p-Chk1 intensity was analyzed in 36 cells (siNeg—HU), 50 cells (siNeg + HU), 35 cells (siCK1δ - HU) and 30 cells (siCK1δ + HU). *p<0.05, **p<0.01. Knockdown of CK1δ in this experiment was confirmed by western blotting (data not shown).

To further evaluate the impact of CK1δ on Chk1 function, we examined the intracellular distribution of Chk1 in MEF^*Ctl*.^ cells treated with CK1δ or negative control siRNA. Typically, Chk1 localizes to the cytoplasm and nucleus; retention in the nucleus requires Ser345 phosphorylation [28]. As expected, in cells transfected with negative control siRNA total Chk1 was detected in the cytoplasm and nucleus. Exposure to HU increased the relative signal intensity in the nucleus, consistent with Chk1 activation (Fig 7A). The accumulation of Chk1 in the nucleus was particularly striking when cells were stained with an antibody directed against an epitope containing phosphorylated Ser345 (Fig 7B). In contrast, the nuclei of cells transfected with CK1δ siRNA were largely devoid of total Chk1 regardless of exposure to HU (Fig 7A). HU treatment stimulated a discernible accumulation of phosphorylated Chk1 in nuclei, though the signal was much weaker than that seen in the negative control (Fig 7B). These findings provided additional evidence that loss of CK1δ expression impaired the ability of Chk1 to mediate repair of DNA damage.

### Chk1 protein stability is decreased after siRNA knockdown of CK1δ

**Fig 8:**
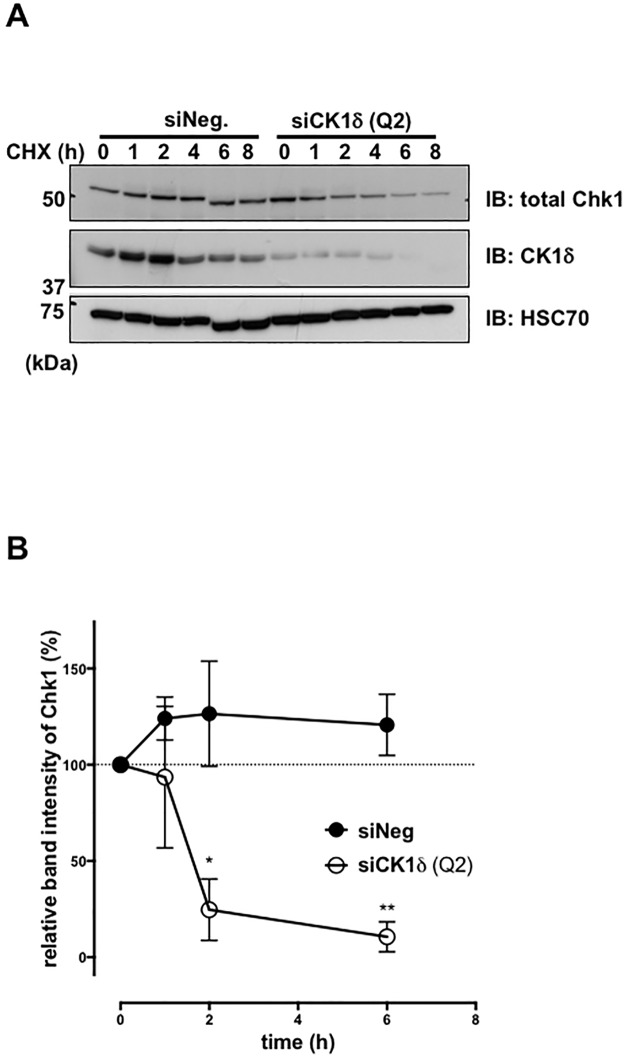
Chk1 protein stability is decreased after siRNA knockdown of CK1δ. (A) Western blot analysis of Chk1 and CK1δ in MEF^*Ctl*.^ cells treated with siNeg or siCK1δ and cycloheximide (CHX, 10 μg/ml) for the indicated times. HSC70 was a loading control. (B) Graph indicates the relative band intensity of Chk1 normalized to loading control in cells treated with the indicated siRNA reagents. Each point is the mean +/- SD of data from three experiments. *p<0.05, **p<0.01.

The reduced amount of Chk1 often seen in cells lacking CK1δ expression suggested that the stability of Chk1 protein is diminished in the absence of CK1δ. To examine Chk1 protein stability, MEF^*Ctl*.^ cells were transfected with either control or CK1δ siRNA, and then treated with cycloheximide (CHX). The Chk1 protein level was intact for at least 6 h after CHX treatment in cells that received the control siRNA. In contrast, the Chk1 protein level decreased significantly in MEF^*Ctl*.^ cells transfected with CK1δ siRNA (Fig 8), indicating that CK1δ contributes to Chk1 protein stability.

## Discussion

This study demonstrated that loss of CK1δ expression in mouse cells results in genomic instability and the accumulation of DNA damage. The former was manifested by grossly enlarged nuclei in embryonic retinal tissue and multiple abnormal phenotypes in cultured MEF cells, including multiple centrosomes, micronuclei and aneuploidy documented by flow cytometry. These findings were consistent with earlier work in yeast that linked the disruption of the CK1δ ortholog to genomic instability and aberrant DNA repair [8, 9]. Previous experiments with human trophoblasts involving the use of the CK1δ/ε kinase inhibitor IC261 also implicated CK1δ in mediating normal spindle assembly and function [42, 43]. However, the interpretation of these data was challenged by subsequent reports claiming that IC261 bound directly to tubulin and thereby disrupted the mitotic spindle [44, 45]. Several articles have linked CK1δ to the regulation of p53 turnover via multiple mechanisms in the context of DNA damage [14–18]. Our work suggests there are other mechanisms for CK1δ function in DNA repair that involve the regulation of Chk1 activation and protein stability. In the absence of CK1δ, basal levels of Chk1 protein typically were reduced and activation in response to HU-induced replicative stress was significantly diminished. A corresponding decline of activated Chk1 in the nucleus we presume contributed to a defective DDR.

A full account of the mechanisms responsible for CK1δ regulation of Chk1 remains to be determined. Considering the many substrates and cellular processes affected by CK1δ, a combination of direct and indirect mechanisms probably are responsible for the decline in Chk1 activation and stability in the absence of CK1δ. Furthermore, our experiments designed to test the relevance of CK1δ kinase activity for the regulation of Chk1 yielded ambiguous results. The inability of a CK1δ kinase-dead construct to rescue the CK1δ siRNA-dependent decline in phosphorylated Chk1 implied that CK1δ catalytic activity was important (Fig 6). However, the CK1δ kinase inhibitors, PF670462 and LH846, did not have a significant effect on Chk1 phosphorylation (S6 Fig). One possible explanation for this discrepancy relates to the timing of kinase inhibition: in the siRNA experiments, the cells lacked CK1δ catalytic activity for days prior to HU addition, whereas cells were pre-incubated with the kinase inhibitors only 1 h prior to the 1.5 h treatment with HU. We surmise that the prolonged suppression of CK1δ expression and catalytic activity in the siRNA rescue experiment accounted for the decrease in phosphorylated Chk1. It is also possible that the kinase inhibitors disrupt the activity of other enzymes that offset the impact of CK1δ inhibition. Alternatively, the CK1δ kinase-dead derivative might have been inactive because it failed to bind a critical partner, rather than because it was deficient in kinase activity. However, our co-immunoprecipitation experiments demonstrated that the interactions of Chk1 with wild type and kinase-dead CK1δ were similar (S7 Fig), making this explanation less likely.

The relative levels of Chk1 protein in MEF cells expressing normal vs. reduced amounts of CK1δ varied substantially, suggesting that multiple factors were involved in determining Chk1 protein levels, and their contributions varied in different experiments. Chk1 phosphorylation was diminished in cells lacking CK1δ even when there was little change in total Chk1, implicating additional mechanisms in the dysregulation of Chk1 activation. We confirmed a previous report that CK1δ and Chk1 co-immunoprecipitate with each other [32] (S7 Fig). We infer that the association of CK1δ and Chk1 protects the latter from proteolytic degradation. Chk1 undergoes ubiquitin-dependent, proteasomal degradation that is mediated by E3 ubiquitin ligase complexes, including F-box Protein (Fbx6)-containing SCF (Skp1-Cul1-F-box) E3 ligase [46], cullin protein Cul4A/DNA-binding protein 1 (DDB1) [47] and X-linked inhibitor of apoptosis (XIAP)-XIAP-associated factor 1 (XAF1) complex [48]. Our attempts to evaluate the dependence of Chk1 ubiquitination on CK1δ expression were inconclusive. Future work should address the potential role of CK1δ in regulating Chk1 ubiquitination.

The reduced amount of total and phosphorylated Cdc2/CDK1 in cells lacking CK1δ also raises mechanistic as well as functional questions. Cdc2/CDK1 turnover during genotoxic stress has been attributed to polyubiquitination following its phosphorylation by double stranded RNA-activated protein kinase [49, 50]. We have not explored the possibility that this process is stimulated in the absence of CK1δ. While a decrease in phosphorylated Cdc2/CDK1 would correlate with a failure in the mitotic checkpoint, it is uncertain whether the low concentration of unphosphorylated Cdc2/CDK1 remaining in the cell would be sufficient to enable cell cycle progression through mitosis. Conceivably, a fraction of cells accumulates DNA damage and remain in cell cycle arrest, while others proceed through the cell cycle and undergo mitotic catastrophe or subsequently die as a result of accumulated genotoxic damage.

Other members of the CK1 family have been implicated in the regulation of Chk1 activation and Cdc2/CDK1 phosphorylation. CK1γ1 phosphorylation of Claspin, a binding partner of Chk1, is critical for Chk1 activation, but CK1γ1 has little or no effect on total Chk1 protein level [51]. CK1α phosphorylates Cdc25, priming it for β-TrCP-dependent, ubiquitin-mediated proteolysis [52]. Because Cdc25 is responsible for removing the phosphate group from Tyr15 in Cdc2/CDK1, loss of CK1α would result in dephosphorylation of Cdc2/CDK1 and consequently its activation. In the study by Honaker and Piwnica-Worms, siRNA knockdown of CK1δ, CK1ε and CK1γ1 had no effect on Cdc25 stability [52]. A subsequent publication contained evidence that CK1ε did phosphorylate Cdc25, and thereby promoted its degradation [53]. Perhaps the discrepancy in these two reports relates to differences in their respective cellular models (HeLa in the former, 293 in the latter). Combined with our findings, these articles indicate that the CK1 family regulates key effectors of the DDR and cell cycle checkpoints in various ways that likely are cell context specific.

The presence of cells with abnormally enlarged nuclei indicative of aneuploidy in the retina of Csnk1δ null mouse embryos suggested that the reduced size of organs in perinatal null mice might be due at least in part to defects in genomic stability, DNA repair and cell cycle checkpoints. We speculate that homeostatic mechanisms would not fully overcome the inordinate amount of cell death attributable to these defects. Detailed analysis of the perinatal lethal phenotype was beyond the scope of this project. A limited number of autopsies indicated that the overall appearance of major organs was intact. However, one 18.5-day old *Csnk1d* null embryo showed signs of microcephaly (S8 Fig) and others had features suggestive of focal CNS abnormalities (S9 Fig). Recently, deletion of the human CSNK1D gene has been associated with a rare incidence of microcephaly and cardiovascular malformation [54]. Such abnormalities have been observed in patients with ciliopathies [55–58], and as we previously reported, CK1δ has a role in ciliogenesis [6]. Given the many processes that are affected by CK1δ, including neurite outgrowth [3, 5], perinatal lethality might be a consequence of one or more deficiencies. It should be noted that the perinatal lethal phenotype was dependent on genetic background. Clearly, the full impact of CK1δ loss was influenced by other genetic factors. Similarly, we observed compensation or adaptation of MEF^*Csnk1d null*^ cells in culture, as a massive amount of cell death in early passages was followed by a gradual decrease in the percentage of cell death with additional passaging. Nonetheless, the defect in Chk1 protein level persisted at least through passage 20.

## Conclusions

The loss of CK1δ expression in mammalian cells results in genomic instability and the accumulation of DNA damage. Abrogation of CK1δ expression also increased the vulnerability of cells to genotoxic stress. Inhibition of CK1δ expression was associated with a decline in total and phosphorylated Chk1, an important mediator of DNA damage repair and mitotic checkpoints, suggesting that regulation of Chk1 by CK1δ contributes to the proper functioning of these processes.

## Supporting Information

S1 Fig Gross appearance of *Csnk1d* knockout mouse. All pups were collected within 24 h after birth.
(PDF)

S2 Fig MEF^*Csnk1d null*^ cells exhibits higher sub-G0/G1 population compared with MEF^Ctl.^ Cells. Cell cycle analysis was performed with PI staining using different passage number of MEF^*Ctl*.^ cells and MEF^*Csnk1d null*^ cells. Red peaks correspond to G0/G1 and aqua peaks correspond to sub G0/G1 region. The latter is comprised of dead cells or cell fragments with markedly reduced amounts of DNA.
(PDF)

S3 Fig Lack of CK1δ elicits DNA damage response in MCF7 human breast cancer cells. (A) Cells were transfected with negative control siRNA (siNeg) or two different siRNAs targeting CK1δ (siCK1δ Q6 and siCK1δ Am). 72 h later, cells were collected and lysates were immunoblotted for γ-H2AX. Panel represents one of three independent experiments. (B) Relative band intensity of γ-H2AX normalized to loading control. Data are presented as the mean plus standard deviation of three experiments. *p<0.05. (C) Cells that had been treated as described in (A) were immunostained with DAPI and γ-H2AX antibody. Bars, 20 μm. (D) Relative fluorescence intensity of γ-H2AX. Six images in each group (siNeg, siCK1δ Q6, siCK1δ Am) were analyzed with Image J software. **p<0.01, ***p<0.001.
(PDF)

S4 Fig CK1δ /ε inhibitor induces DNA damage in MEF^*Ctl*^ cells. (A) MEF^*Ctl*^ cells were treated with PF670462 (10 μM) for 5 hours. Scale bar = 10 μm. (B) Micronuclei formation was induced by PF670462 treatment in MEF^*Ctl*^ cells. Incidence of micronuclei was measured in control (DMSO) and PF670462 treated groups; 50 and 54 cells were counted respectively, and statistical analysis was performed with Fisher’s exact test. ***p<0.0001. (C) Cell viability was tested with MTS assay. MEF^*Ctl*^ cells were treated with PF670462 for 3 days. Data is shown as average of 4 independent experiments with mean +/- SD. **p<0.01, ***p<0.001 compared with DMSO control.
(PDF)

S5 Fig CK1δ siRNA knockdown in MCF7 cells decreases basal Chk1 protein level and Chk1 phosphorylation following HU treatment. (A) Cells were transfected with negative control or CK1δ siRNA and treated with HU for the indicated times. Cell lysates were immunoblotted as indicated. (B) Bar graph shows the relative intensity of bands in (A) that were normalized to HSC70. Data are from one representative experiment of multiple experiments.
(PDF)

S6 Fig Two CK1δ/ε kinase inhibitors did not block HU-induced Chk1 activation in MEF^*Ctl*^ cells. MEF^*Ctl*^ cells were pre-incubated with the indicated concentrations of PF670462 or LH846 for 1 h, subsequently treated with HU for 1.5 h and harvested for western blotting.
(PDF)

S7 Fig CK1δ is associated with Chk1 via its kinase domain. HEK293 cells were transfected with FLAG-Chk1 and various Myc-CK1δ derivatives (FL: CK1δ full length, K38A: kinase inactive mutant; KD: kinase domain only; CT: carboxy-terminus only) [6]. 48 h later, cells were lysed, immunoprecipitated with FLAG antibody and immunblotted as indicated.
(PDF)

S8 Fig *Csnk1d* null embryo (E18.5) showed abnormalities in the brain. *Csnk1d* null embryo #A: The cranial vault is greatly expanded compared to the WT. The brain appeared compressed both dorsally and ventral. Throughout the midbrain and brainstem and in the cortical plate are foci of hemorrhage and necrosis. The subventricular zone in the forebrain appears thickened and disorganized compared to WT. The 4^th^ ventricle, aqueduct and lateral ventricle are more dilated than in the WT. *Csnk1d* null embryo #B: Possible mild compression compared to the WT. In the forebrain, possible increased streaming of subventricular cells into the intermediate zone.
(PDF)

S9 Fig Brain histology in Csnk1δ null embryo (E18.5). Area 1 shows pontomedullary/medullary hindbrain, and Area 2 shows midbrain stained with H&E. Note that at higher magnification, cells were detected in Csnk1δ null embryos with large cell/nuclear size and abnormal cell shape compared with cells in WT tissue.
(PDF)

S1 Table Antibodies used for immunblotting and immunostaining, and siRNA reagents used in this study. (PDF)

## Abbreviations

Chk1: Checkpoint kinase 1
CK1: casein kinase 1
MEF: mouse embryonic fibroblast
WT: wild type
