# Supplementary figures and images for: Lack of Casein Kinase 1 Delta Promotes Genomic Instability - The Accumulation of DNA Damage and Down-Regulation of Checkpoint Kinase 1
Source: PLoS One. 2017 Jan 26;12(1):e0170903. doi: 10.1371/journal.pone.0170903 (PMC5268481; doi:10.1371/journal.pone.0170903)

**S1 Fig**

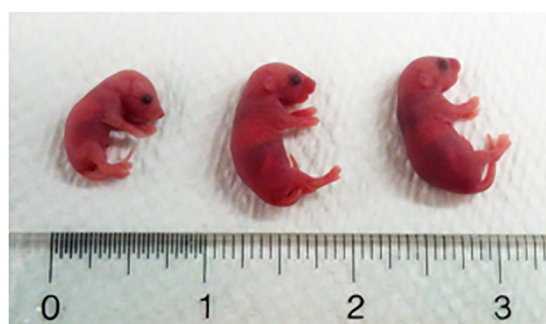

**CSNK1D  
Genotyping  
Result**

**(-/-)**

**(+/-)**

**(+/-)**

Supplement: S1 Fig — All pups were collected within 24 h after birth. (PDF) [file pone.0170903.s001.pdf]

## S2 Fig

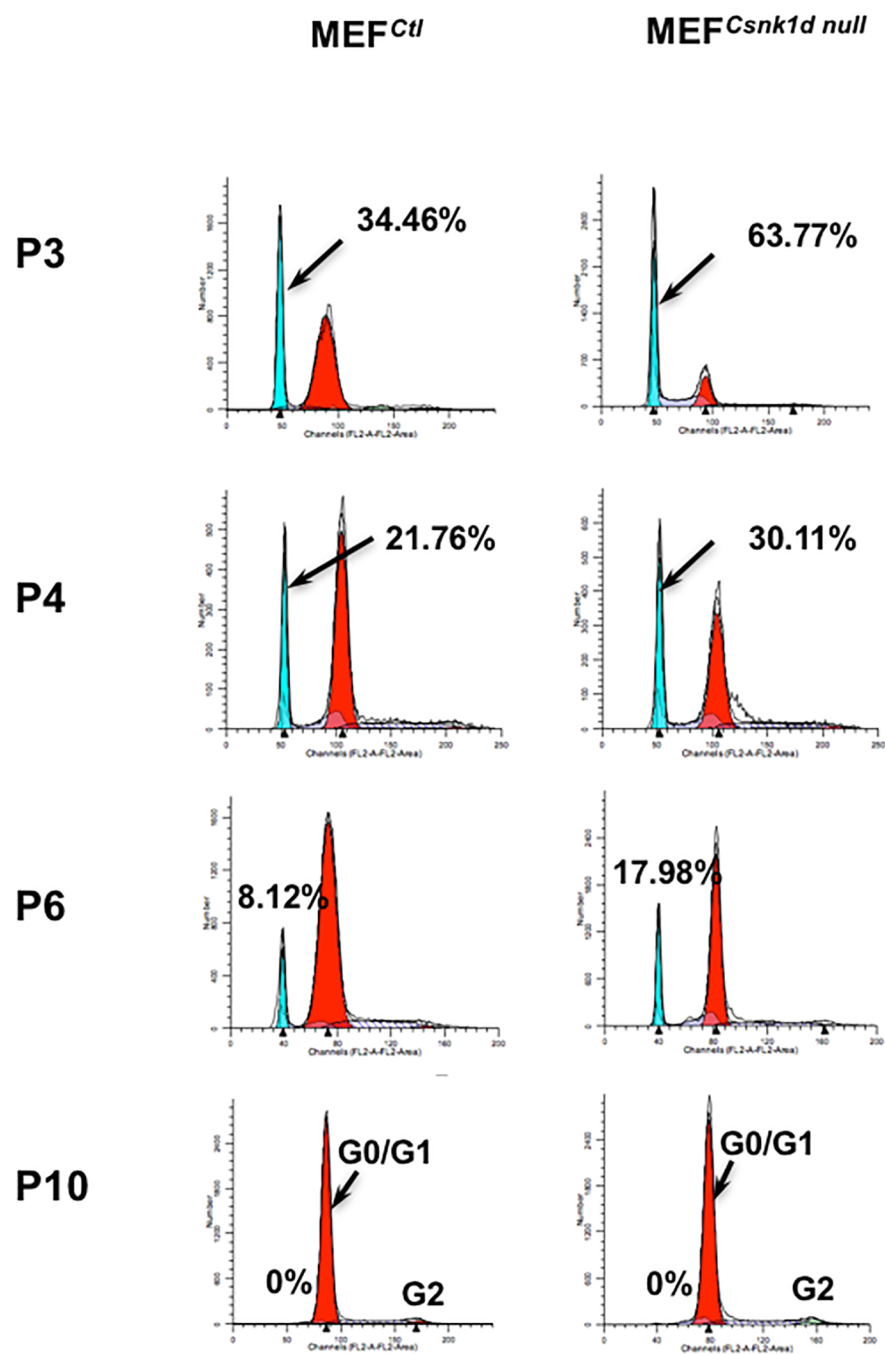

Supplement: S2 Fig — Cell cycle analysis was performed with PI staining using different passage number of MEFCtl. cells and MEFCsnk1d null cells. Red peaks correspond to G0/G1 and aqua peaks correspond to sub G0/G1 region. The latter is comprised of dead cells or cell fragments with markedly reduced amounts of DNA. (PDF) [file pone.0170903.s002.pdf]

**S3 Fig**

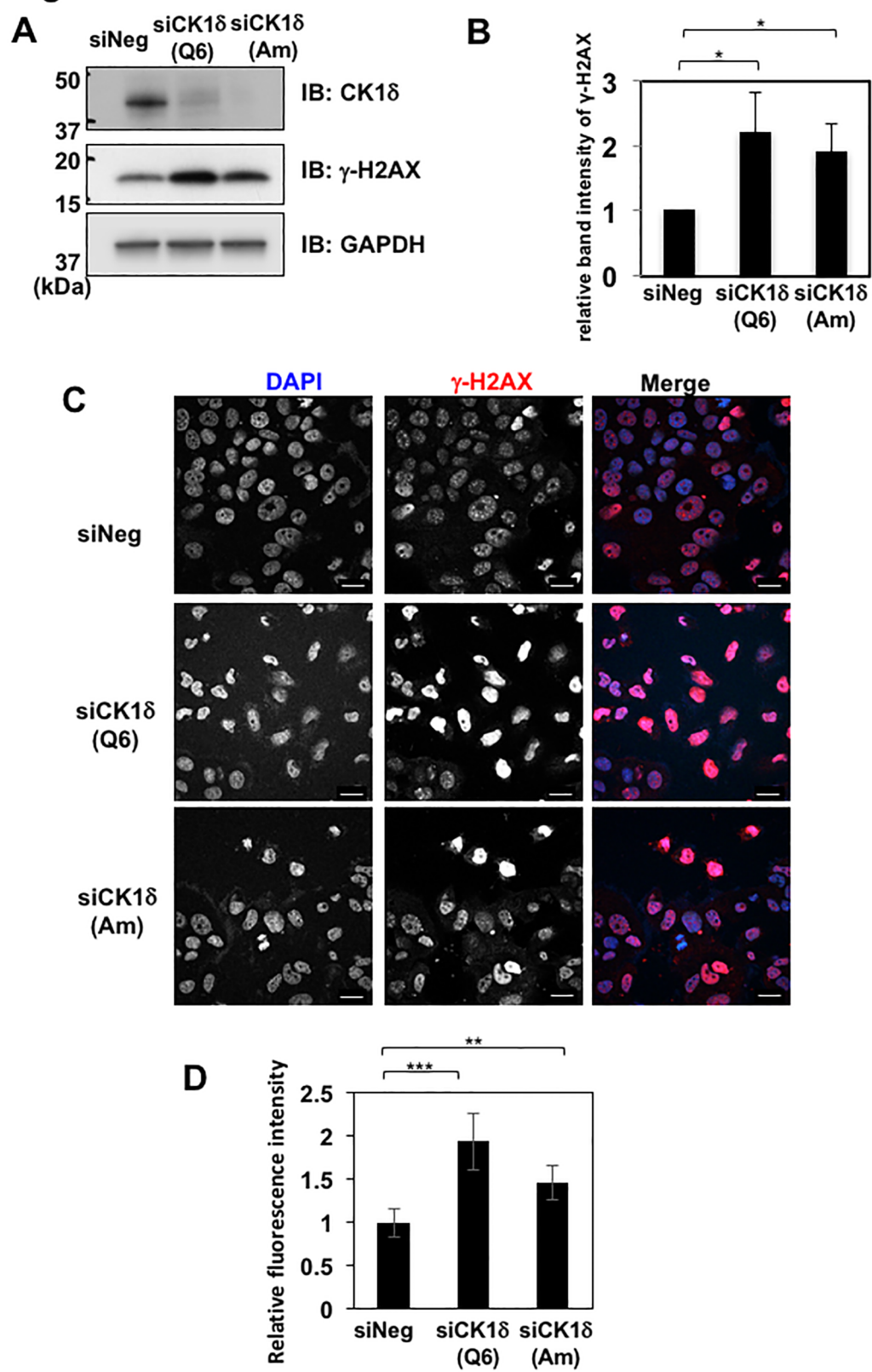

Supplement: S3 Fig — (A) Cells were transfected with negative control siRNA (siNeg) or two different siRNAs targeting CK1δ (siCK1δ Q6 and siCK1δ Am). 72 h later, cells were collected and lysates were immunoblotted for γ-H2AX. Panel represents one of three independent experiments. (B) Relative band intensity of γ-H2AX normalized to loading control. Data are presented as the mean plus standard deviation of three experiments. *p<0.05. (C) Cells that had been treated as described in (A) were immunostained with DAPI and γ-H2AX antibody. Bars, 20 μm. (D) Relative fluorescence intensity of γ-H2AX. Six images in each group (siNeg, siCK1δ Q6, siCK1δ Am) were analyzed with Image J software. **p<0.01, ***p<0.001. (PDF) [file pone.0170903.s003.pdf]

S4 Fig

A

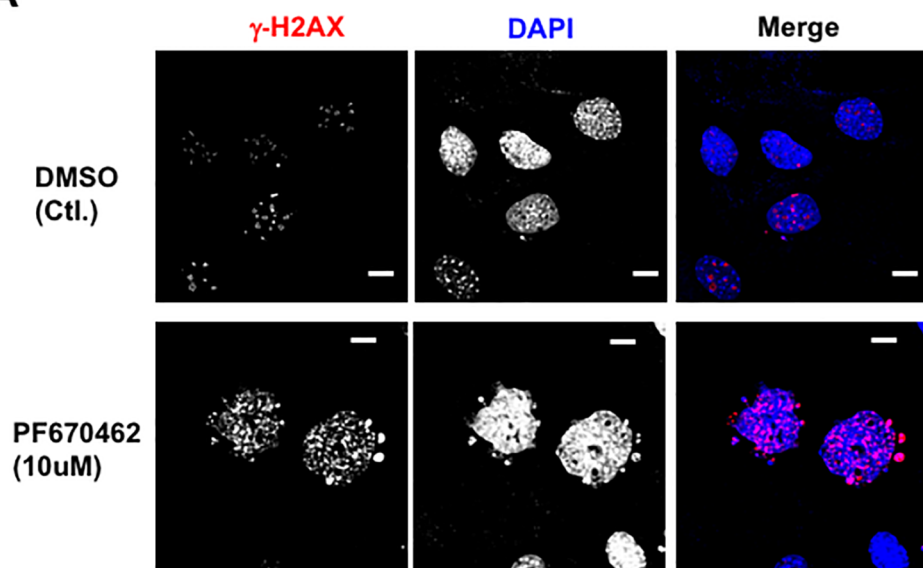

B

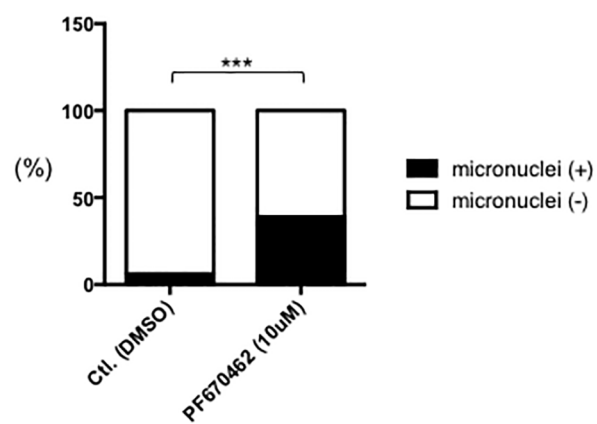

C

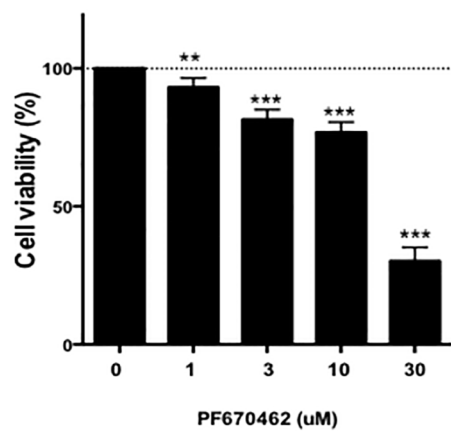

Supplement: S4 Fig — (A) MEFCtl cells were treated with PF670462 (10 μM) for 5 hours. Scale bar = 10 μm. (B) Micronuclei formation was induced by PF670462 treatment in MEFCtl cells. Incidence of micronuclei was measured in control (DMSO) and PF670462 treated groups; 50 and 54 cells were counted respectively, and statistical analysis was performed with Fisher’s exact test. ***p<0.0001. (C) Cell viability was tested with MTS assay. MEFCtl cells were treated with PF670462 for 3 days. Data is shown as average of 4 independent experiments with mean +/- SD. **p<0.01, ***p<0.001 compared with DMSO control. (PDF) [file pone.0170903.s004.pdf]

**S5 Fig**

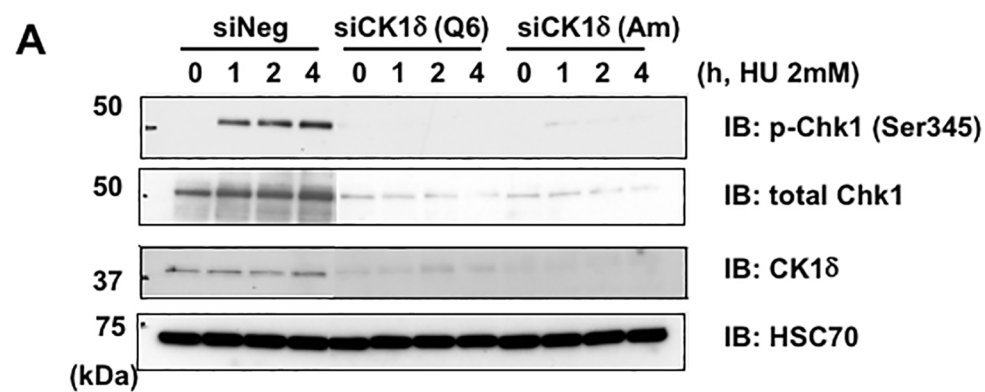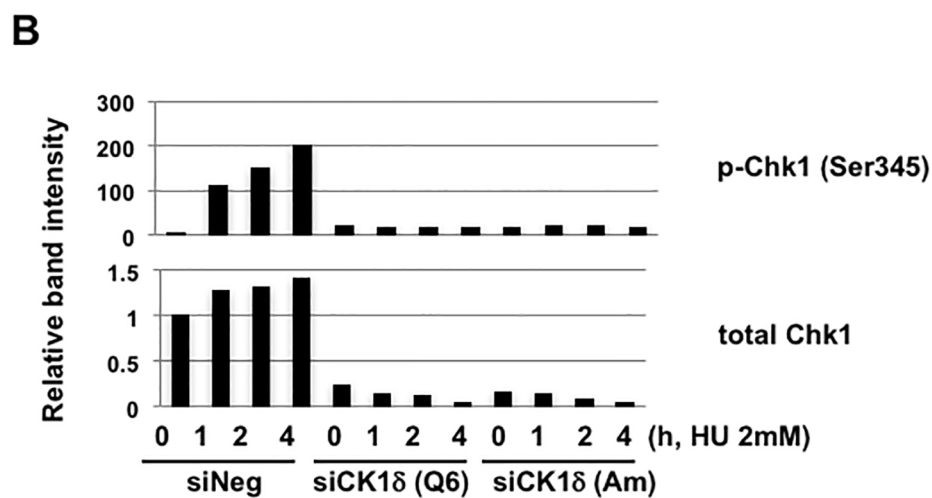

Supplement: S5 Fig — (A) Cells were transfected with negative control or CK1δ siRNA and treated with HU for the indicated times. Cell lysates were immunoblotted as indicated. (B) Bar graph shows the relative intensity of bands in (A) that were normalized to HSC70. Data are from one representative experiment of multiple experiments. (PDF) [file pone.0170903.s005.pdf]

S6 Fig

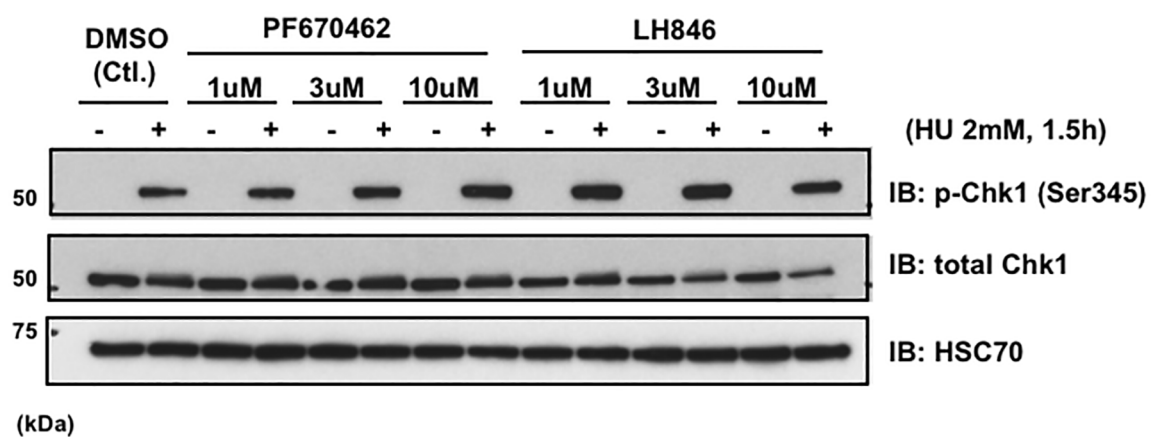

Supplement: S6 Fig — MEFCtl cells were pre-incubated with the indicated concentrations of PF670462 or LH846 for 1 h, subsequently treated with HU for 1.5 h and harvested for western blotting. (PDF) [file pone.0170903.s006.pdf]

S7 Fig

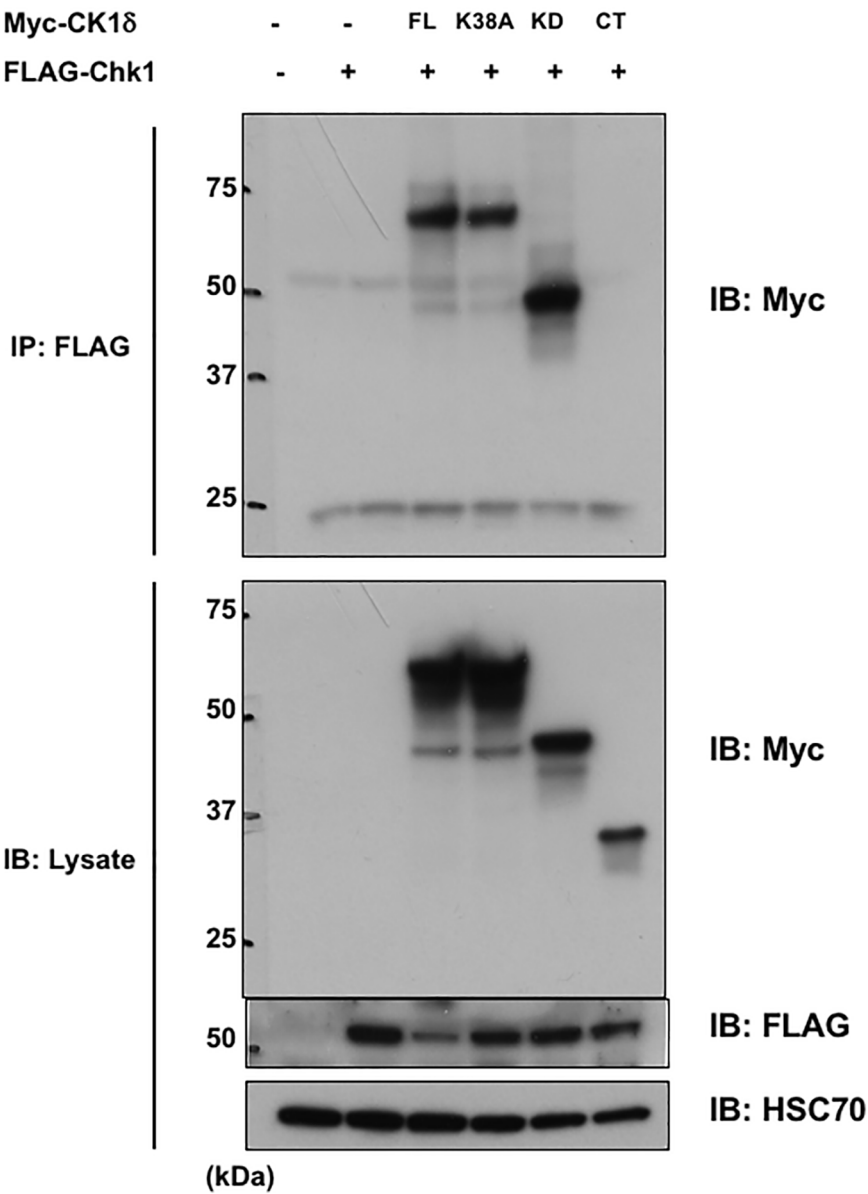

Supplement: S7 Fig — HEK293 cells were transfected with FLAG-Chk1 and various Myc-CK1δ derivatives (FL: CK1δ full length, K38A: kinase inactive mutant; KD: kinase domain only; CT: carboxy-terminus only) [6]. 48 h later, cells were lysed, immunoprecipitated with FLAG antibody and immunblotted as indicated. (PDF) [file pone.0170903.s007.pdf]

**S8 Fig**

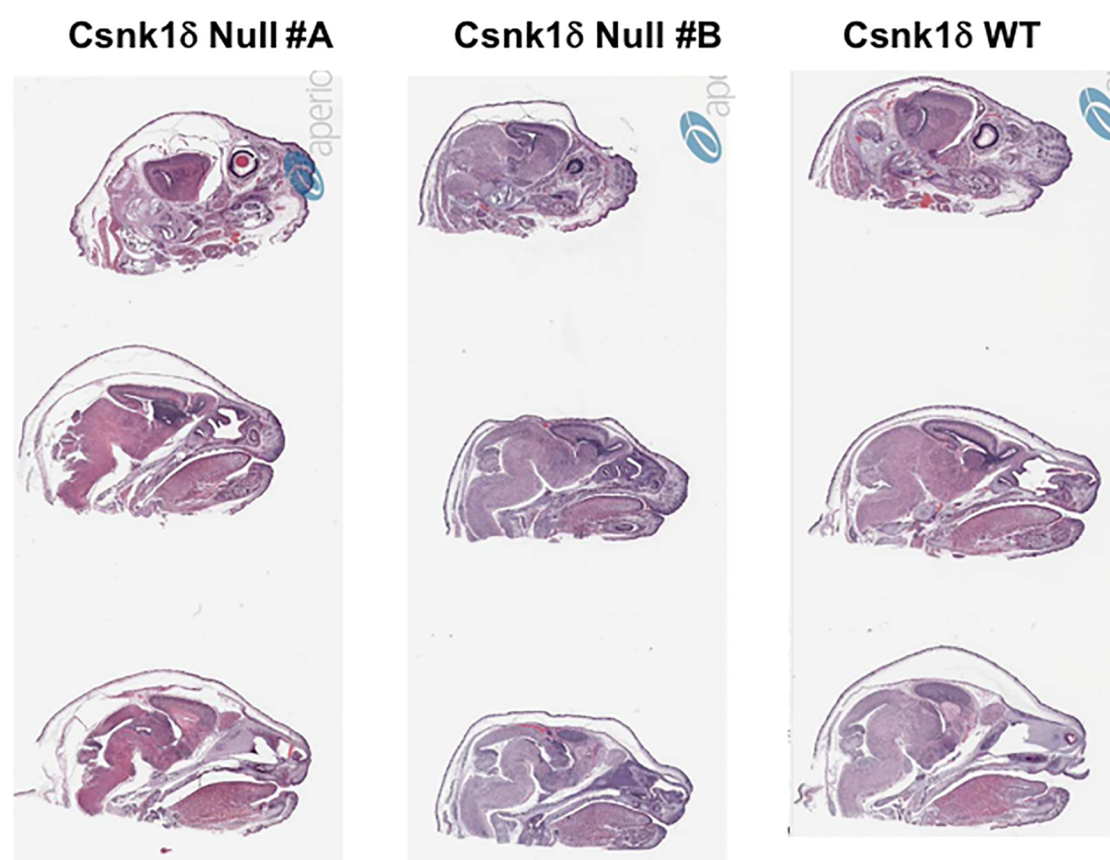

Supplement: S8 Fig — Csnk1d null embryo #A: The cranial vault is greatly expanded compared to the WT. The brain appeared compressed both dorsally and ventral. Throughout the midbrain and brainstem and in the cortical plate are foci of hemorrhage and necrosis. The subventricular zone in the forebrain appears thickened and disorganized compared to WT. The 4th ventricle, aqueduct and lateral ventricle are more dilated than in the WT. Csnk1d null embryo #B: Possible mild compression compared to the WT. In the forebrain, possible increased streaming of subventricular cells into the intermediate zone. (PDF) [file pone.0170903.s008.pdf]

## S9 Fig

### Area1: PMH/MH (pontomedullary/medullary hindbrain)

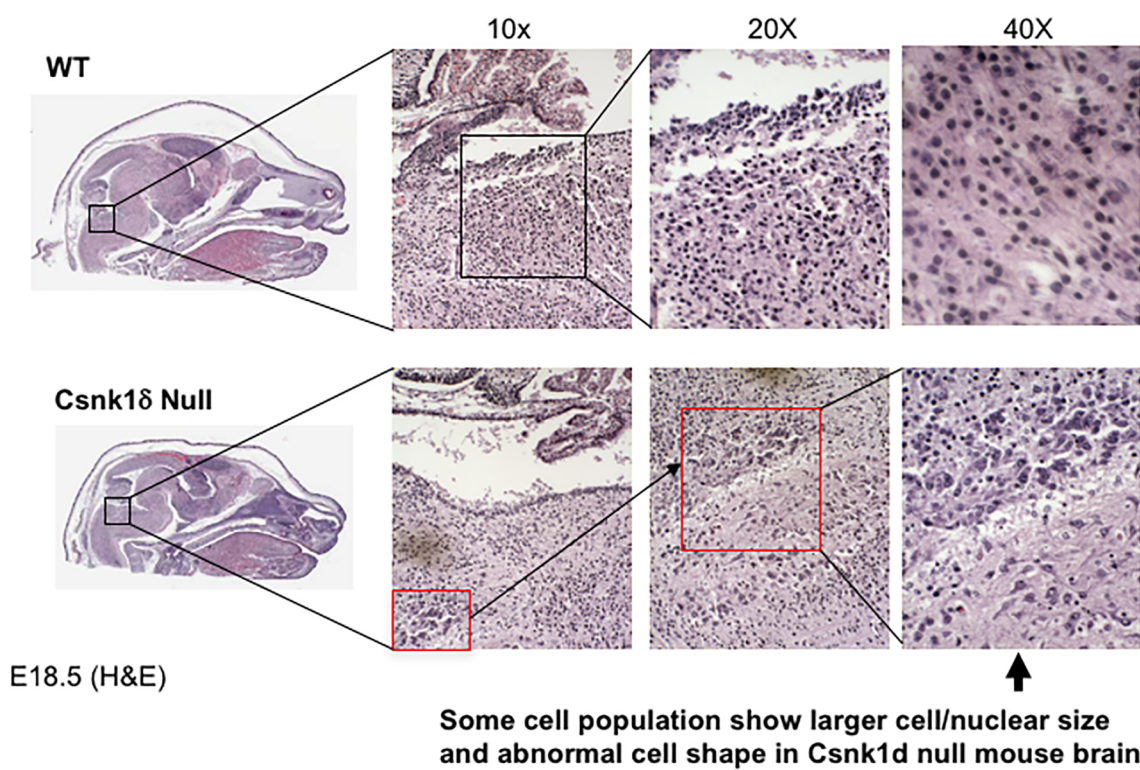

### Area2: Midbrain

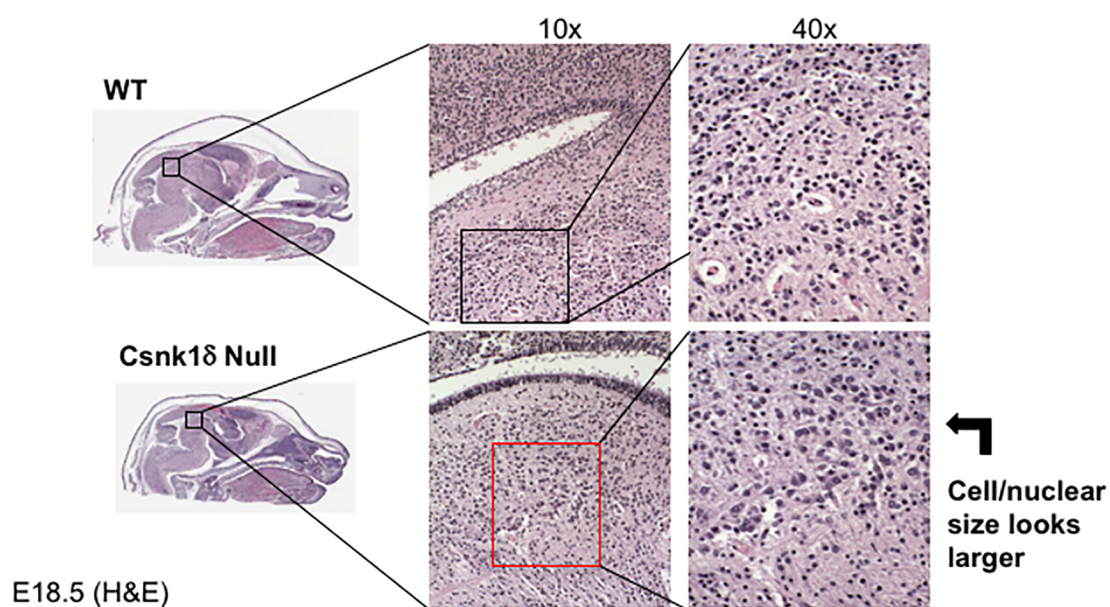

Supplement: S9 Fig — Area 1 shows pontomedullary/medullary hindbrain, and Area 2 shows midbrain stained with H&E. Note that at higher magnification, cells were detected in Csnk1δ null embryos with large cell/nuclear size and abnormal cell shape compared with cells in WT tissue. (PDF) [file pone.0170903.s009.pdf]
