# Supplementary material for: Lack of Casein Kinase 1 Delta Promotes Genomic Instability - The Accumulation of DNA Damage and Down-Regulation of Checkpoint Kinase 1
Source: PLoS One. 2017 Jan 26;12(1):e0170903. doi: 10.1371/journal.pone.0170903 (PMC5268481; doi:10.1371/journal.pone.0170903)
Supplement: S1 Table — (PDF) [file pone.0170903.s010.pdf]

S1 Table

antibody	host species	vendor/source	catalog number	IF (dilution)	WB (dilution)
acetylated-tubulin	Mouse	Sigma	T6793	IF (1:2000)	N/A
gamma-tubulin	Rabbit	Sigma	T3559	IF (1:2000	N/A
gamma-tubulin	Goat	Santa Cruz	sc-7396	IF (1:500)	N/A
gamma-H2AX	Mouse	EMD Millipore	05-636	IF (1:5000)	WB (1:5000)
gamma-H2AX	Goat	Santa Cruz	sc-7396	IF (1:500)	N/A
CK1delta	Mouse	Santa Cruz	sc-55553	N/A	WB (1:500)
Chk1	Mouse	Santa Cruz	sc-8408	IF (1:500)	WB (1:1000)
Phospho-Chk1 (Ser345)	Rabbit	Cell Signaling	#2348	IF (1:250)	WB (1:500)
Cdc2/CDK1	Rabbit	GeneTex	GTX108120	N/A	WB (1:500)
phospho-Cdc2/CDK1 (Tyr15)	Rabbit	Cell Signaling	#4539	N/A	WB (1:500)
HSC70	Mouse	Santa Cruz	sc-7298	N/A	WB (1:5000)
Myc	Mouse	Life Technologies	R950-25	N/A	WB (1:2000)
alpha-tubulin	Mouse	Life Technologies	A11126	N/A	WB (1:5000)
LC3 A/B	Rabbit	Cell Signaling	#4108	IF (1:250)	N/A
LAMP1	Mouse	Santa Cruz	sc-17768	IF (1:250)	N/A

siRNA	Vendor/source	cat#	sense sequence
Mm_Csnk1d_1 FlexiTube siRNA	Qiagen	SI00960883	CAAUUGUGUAAAUAAUAAAtt
Mm_Csnk1d_2 FlexiTube siRNA	Qiagen	SI00960890	GAAGGAUGUUGACCUUAAAtt
Hs_CSNK1D_6 FlexiTube siRNA	Qiagen	SI00287413	CCCUGACGAUUCCACUGUAtt
Human CSNK1D siRNA	Ambion	4392420	UGAUCAGUCGCAUCGAAUAtt
All Stars Negative Control siRNA	Qiagen	SI0365038	N/A
